# Comparative Evaluation of AmnioGuard and Advanced Platelet-Rich Fibrin When Combined With NovaBone Putty in the Regeneration of Human Periodontal Infrabony Defects: Protocol for a Randomized Controlled Clinical Trial

**DOI:** 10.2196/75987

**Published:** 2026-02-26

**Authors:** Geeta Bhandari, Priyanka Jaiswal, Shweta Bhagat, Sakshi Kotecha

**Affiliations:** 1 Sharad Pawar Dental College and Hospital, Datta Meghe Institute of Higher Education and Research (DMIHER(DU)), Sawangi, Wardha, 442001, India, 91 8237719188

**Keywords:** periodontal regeneration, bone graft biomaterials, guided tissue regeneration, platelet-derived growth factors, clinical periodontology, osteoconductive materials, wound healing modulation, leukocyte-rich fibrin, probing depth reduction, attachment level gain

## Abstract

**Background:**

Periodontitis causes progressive attachment loss, which in turn causes tooth mobility and, ultimately, tooth loss. Regenerating and rebuilding the lost periodontal tissue is the goal of periodontal regenerative surgery. Regeneration using AmnioGuard, advanced platelet-rich fibrin (A-PRF), and NovaBone Putty has been demonstrated to be successful in increasing clinical attachment level and decreasing probing pocket depth.

**Objective:**

This study aims to compare and evaluate the effectiveness of AmnioGuard and A-PRF membrane in combination with NovaBone Putty in the regeneration of human periodontal infrabony defects.

**Methods:**

A total of 24 patients will be selected and divided into 2 groups: group A (n=12, 50%) and group B (n=12, 50%). Group A will receive treatment with NovaBone Putty and AmnioGuard, whereas group B will receive treatment with autologous A-PRF and NovaBone Putty. Plaque index, clinical attachment level, probing pocket depth, complete mouth bleeding score, and intraoral periapical radiographs will be statistically analyzed. Clinical and radiographic features will be evaluated before surgery and 6 months following the procedure.

**Results:**

Recruitment began on September 16, 2025, and is ongoing. As of manuscript submission, 4 participants have been enrolled. Results are expected to be published in 2027. All the clinical measurements will be recorded after 6 months. It can be anticipated that AmnioGuard membrane with NovaBone Putty will provide additional benefits in the management of chronic periodontitis by promoting better healing and clinical outcomes.

**Conclusions:**

Within the constraints of this study, the AmnioGuard membrane combined with NovaBone Putty may be more effective in the treatment of chronic periodontitis.

## Introduction

### Background

A dysbiotic dental biofilm that gradually destroys bone and periodontal attachment is a hallmark of periodontitis, a chronic, multifactorial, inflammatory illness. This condition negatively affects speech, mastication, aesthetics, psychological well-being, and overall quality of life [1]. On the basis of bone resorption patterns, periodontal defects are traditionally classified as supraosseous (suprabony) or infraosseous (infrabony). Goldman and Cohen [2] further categorized infrabony defects by the number and configuration of residual osseous walls surrounding the periodontal pocket. Infrabony defects pose a significant clinical challenge as they require both structural regeneration and the restoration of periodontal function. Regeneration is defined as “the reproduction or reconstitution of a lost or injured part to restore the architecture and function of the periodontium” [3]. To achieve this, regenerative periodontal therapy aims to stimulate the formation of new bone, periodontal ligament, and cementum. NovaBone Putty, a next-generation calcium phosphosilicate material derived from bioactive glass, offers multiple advantages for regeneration. It is osteoconductive, osteostimulative, and biocompatible, with hemostatic and anti-inflammatory effects. Its premixed formulation enhances clinical handling and allows for optimal defect adaptation [4]. AmnioGuard, a human amniotic membrane (AM)–based barrier, has also gained attention due to its anti-inflammatory, antifibrotic, antibacterial, and antiscarring properties. Its high laminin subunit alpha-5 content supports epithelial adhesion and accelerates healing, whereas its role as a biological barrier aids in preventing epithelial downgrowth—an essential aspect of guided tissue regeneration (GTR) [5-7]. In recent years, biological membranes have become increasingly important in regenerative therapies. Since their initial application in skin grafting in 1910, their role has expanded across medical and dental fields. In particular, AmnioGuard has shown potential to reduce microbial penetration and modulate wound healing responses [8]. In addition, advanced platelet-rich fibrin (A-PRF), rich in leukocytes, contributes to regeneration by releasing growth factors such as transforming growth factor beta 1, epidermal growth factor, and insulin-like growth factor, all of which enhance fibroblast proliferation and tissue healing [9]. Although several studies have assessed the individual regenerative potential of NovaBone Putty and AmnioGuard, limited clinical data directly compare their effectiveness in managing periodontal infrabony defects. A study conducted by Hazari et al [10] compared NovaBone Putty and platelet-rich fibrin (PRF) and concluded that, in comparison to NovaBone Putty alone, PRF in conjunction with NovaBone Putty produced more favorable findings in terms of relative attachment level gain and higher reduction in probing pocket depth (PPD). A study by Kothiwale et al [11] in which they assessed and compared the effectiveness of bovine-derived xenogenic bone graft (Bio-Oss) and demineralized freeze-dried bone allograft using AM as GTR determined that, while there was no discernible difference between the two materials, both showed a notable improvement in bone fill and percentage gain. Furthermore, evidence regarding clinical outcomes such as probing depth reduction, attachment level gain, and bone fill remains sparse [12]. Therefore, this study aims to address this gap by directly comparing the clinical and radiographic outcomes of NovaBone Putty in combination with A-PRF or AmnioGuard in the treatment of infrabony periodontal defects.

### Objectives

This study aims to evaluate and compare the effectiveness of AmnioGuard and A-PRF in combination with NovaBone Putty in terms of gain in clinical attachment level (CAL), reduction in PPD, and radiographic bone fill in infrabony defects before surgery and 6 months after surgery.

## Methods

### Ethical Considerations

The Institutional Ethics Committee of Datta Meghe Institute of Higher Education and Research, Sawangi (Meghe), Wardha, Maharashtra, India, approved this study (reference DMIHER(DU)/IEC/2025/507). Participants will be provided with an informed consent form clearly describing the study’s aim, procedures, risks, and benefits and their right to opt out at any time. Consent for publication of data will be obtained and used for all analyses, including secondary analyses. We will confirm that the original consent and institutional review board approval explicitly cover the use of primary and secondary data. Collection of data and entering of the data into the database for screening and randomization will be conducted by the primary investigator. The privacy of prospective and enrolled participants’ personal information will be upheld to ensure confidentiality before, during, and after the trial. We will ensure that the data collected are fully anonymized, with no personally identifiable information retained or linked to any participant. As there are no interventions involved and only a cross-sectional survey will be conducted in which the data will be collected via an interview and anthropometric measurements, participants will not be provided with any compensation in this study. For any images presented in the manuscript, appropriate consent will be obtained from the participants.

### Recruitment

#### Overview

Participants in this clinical trial will be those receiving treatment at the Department of Periodontics and Implantology of Sharad Pawar Dental College, Datta Meghe Institute of Higher Education and Research, or those referred for treatment there who have periodontal pockets larger than or equal to 4 mm in at least 10 permanent teeth. All clinical procedures will be performed there. It is thought that this will enhance trial adherence and recruitment because patients will already be receiving treatment at this location.

#### Inclusion Criteria

The inclusion criteria are as follows: (1) at least 1 or 2 interproximal infrabony osseous defects that may be seen on radiography, with a PPD of at least 5 mm and clinical attachment loss of at least 5 mm after starting treatment; (2) clinical and radiographic measurements showing that the intraosseous component of the lesion is ≥3 mm deep (intraoperative assessment will confirm this); (3) defect radiographic base of at least 3 mm coronal to the tooth’s apex; and (4) keratinized gingiva surrounding the test teeth of at least 3 mm wide to completely hide the deficiency with soft tissue.

#### Exclusion Criteria

The exclusion criteria are as follows: (1) patients with evident localized aggressive periodontitis, (2) individuals with poor oral hygiene (plaque index >1), (3) those who have recently smoked more than 10 cigarettes per day or who have used tobacco products, (4) a tooth that has not had enough endodontic or restorative care, (5) a tooth that has a class III or class IV furcation defect and mobility greater than grade II, (6) a history of periodontal surgical treatment in the specific quadrant of the mouth selected for the study, and (7) women who are pregnant or nursing mothers.

### Sample Size Estimations

The following calculations were performed to estimate sample size:

- Mean PPD for AmnioGuard and NovaBone Putty (μ₁) before: 8.6 (SD 1.17) mm
- Mean PPD for AmnioGuard and NovaBone Putty (μ₂) after: 6.9 (SD 1.10) mm
- The standard normal deviate (*Z* value) corresponding to a 2-sided significance level (α) of .05, which equals a 95% confidence level, is given by (1–α/2)=1.96
- Z_(1–β [β=.05; power=95%]) = 1.64 (Z_(1 − β) represents the standard normal deviate corresponding to the study power. With β set at 0.05 (power=95%), the Z value used was 1.64. This differs from Z_(1 − α/2), which corresponds to the significance level (α = 0.05) used in the sample size calculation).
- Combining the 2 aforementioned equations yields the following: n≥[(Z_[1 – α/2] + Z_[1 – β])^2^ × (σ₁^2^ + σ₂^2^/r)]/(μ₁ – μ₂)^2^
- Replacing the aforementioned values into the equation yields the following: n≥[(1.96 + 1.64)^2^ × (1.17^2^ + 1.10^2^/1)]/(8.6 – 6.9)^2^=12

Hence, group 1 and group 2 require a minimum sample size of 12. The minimum total sample size required is 24 [13].

All data will be tested for normality using the Shapiro-Wilk test and for homogeneity of variance using the Levene test to ensure that the assumptions for parametric tests are met.

### Randomization

A total of 24 participants will be selected and receive a different treatment based on allocation to group A or B. For participants allocated to group A (n=12), NovaBone Putty and AmnioGuard will be placed into the osseous defect, and for participants allocated to group B (n=12), osseous defects will be treated with NovaBone Putty and then A-PRF membrane. Participants will be randomly allocated into the 2 treatment groups (NovaBone Putty, A-PRF, and AmnioGuard) using a computer-generated randomization sequence. The randomization list was created using an online random number generator, ensuring equal distribution of participants across both groups. Allocation concealment will be maintained by an independent clinician who is not involved in the clinical procedures or outcome assessments. Group assignments will be revealed only after patient enrollment and consent, thereby minimizing selection bias.

### Procedure

#### Overview

Participants will be enrolled and randomly assigned to the interventions by the researchers in charge of the therapy application. Outcome evaluations were performed on the study participants in accordance with the described procedures and outcome measures. The 15-mm University of North Carolina (UNC-15) probe, which measures plaque index, bleeding on probing, probing depth, pocket depth, and clinical attachment loss, will be used to evaluate participants’ periodontal clinical characteristics. Every patient will receive lessons on proper brushing technique as well as a demonstration of good oral hygiene practices.

Following a thorough examination and diagnosis, the first course of treatment will involve root planing under local anesthesia, supragingival and subgingival scaling, and recommendations on oral hygiene. If necessary, a coronoplasty will be carried out. Instructions for plaque control will be repeated until the patient’s plaque score is less than 1. Six weeks after the initial therapy is finished, a re-evaluation will be conducted to assess the patients’ reaction to the treatment and confirm the necessity of periodontal surgery.

A specially made occlusal acrylic stent will be used to standardize the probe’s location and angulations. The occlusal stent will be constructed using acrylic on a cast model created from an alginate impression. The occlusal stent covers the occlusal surface of the tooth being treated as well as the occlusal surfaces of at least 1 tooth that is mesially and distally next to the afflicted tooth. The stent will also be expanded to cover the coronal part of the teeth. A reference point (slot) will be made on the stent at the deepest point of the impacted tooth to allow for repeatable periodontal probe placement. The apical border will be linear and serve as a fixed reference point.

Before the surgery, the patients will be told to rinse their mouths for a minute with a 0.2% chlorhexidine gluconate solution. Because the patient will be draped, only the oral cavity will be visible. Asepsis will be maintained during the procedure. Depending on the operation, either nerve block or infiltration anesthesia will be used for the surgical site using a local anesthetic solution consisting of 2% lignocaine and 1:100,000 epinephrine. The standard technique, which involves a periodontal access flap, will start with intracervical (sulcular) incisions performed on the buccal and lingual parts using Bard-Parker number 12 or 15 surgical blades. The incisions will be made as far interproximally as possible to preserve the entire interdental papillae and accomplish primary wound closure. Two teeth will be included in the flap: one mesial and one distal to the tooth associated with the defect. In the event that more access is required, divergent vertical relief incisions will be made one tooth distant from the problem. The full-thickness (mucoperiosteal) flap will be mirrored using a periosteal elevator (24G; Hu-Friedy) to reveal the alveolar bone in the area of the osseous defect. Extreme caution will be taken to avoid flap rupture or papillae loss when removal of granulomatous tissue from the inner aspect of the flap occurs. To create autologous PRF, the protocol calls for obtaining 6 to 10 mL of venous blood from the patient’s forearm using an 18-gauge needle in a sterile plastic container. The blood will then be transferred to new, sterile test tubes without the addition of any anticoagulant, and the initial volume of blood will be split equally between 2 tubes that are positioned symmetrically around the rotor axis for proper balancing. The tubes will then be centrifuged for 10 minutes at 3000 revolutions per minute (rpm) to create autologous PRF.

#### Procedure for the Intervention Group

The defect will be completely isolated and hemostatized. To enable quick flap closure following the graft material implantation, the flap will be presutured without tying the knot. With minimal pressure, NovaBone Putty and AmnioGuard will be inserted into the test site’s osseous defect until the flap is raised, filling it up to the osseous wall’s highest coronal level. To ensure primary flap closure, the flap will be coronally adjusted and sutured so that the flap edge is positioned 1 to 2 mm coronal to the cement-enamel junction.

#### Procedure for the Control Group

Except for packing osseous defects at control sites with NovaBone Putty and covering the bone graft with A-PRF membrane, the surgical procedure for the control group will be the same as the procedure for the intervention group.

### Statistical Analysis

A calculation of means and SDs will be conducted, and the mean data will be analyzed using a standard statistical method to determine statistical significance. Unpaired and paired 2-tailed *t* tests will be used to evaluate data at baseline and 6 months for each group. A *P* value greater than .05 will be considered not statistically significant, whereas a *P* value of less than .05 will be deemed significant. The results will be calculated using the SPSS software (version 17; IBM Corp). Group A and group B will be compared based on clinical outcomes of plaque index, full-mouth bleeding score, PPD reduction, and CAL gain using an unpaired *t* test.

### Evaluation Outcomes

The primary outcome of this study is radiographic bone fill at 6 months after surgery. This outcome is crucial as it indicates the extent of bone regeneration achieved through the different treatment modalities being tested. The secondary outcomes are PPD reduction, plaque index, papillary bleeding index, and CAL gain.

PPD reduction, which is used to evaluate the decrease in the depth of periodontal pockets, a sign of improved periodontal health, will be measured using an acrylic stent and a UNC-15 probe. Once the acrylic stent is in place, the UNC-15 probe will be positioned at the appropriate angle to access the deepest part of the interproximal pocket within the crevicular space. The point at which the probe makes contact with the stent will be marked in pencil. This will provide a consistent point of reference for subsequent measurements.

Plaque index will be calculated using the Turesky-Gilmore-Glickman modification of the Quigley-Hein index.

Papillary bleeding index will be calculated using the Muhlemann-Son Sulcus Bleeding Index.

CAL gain, which measures the improvement in the attachment of the periodontal tissue to the tooth, indicating successful regeneration, will be measured using a UNC-15 probe positioned at the base of the pocket.

## Results

Ethical approval was obtained from the Institutional Ethics Committee of Datta Meghe Institute of Higher Education & Research on February 7, 2025. Recruitment began on September 16, 2025, and is ongoing. As of manuscript submission, 4 participants have been enrolled. Data collection is ongoing, and data analysis has not yet been completed. Results are expected to be published in 2027.

All the clinical measurements will be recorded at baseline and 6 months. Plaque index, which indicates the degree of full-mouth supragingival plaque accumulation, will be used to assess the patient’s dental hygiene status and will be measured at the 1-month follow-up. Although a substantial difference between the groups is not anticipated, AmnioGuard and NovaBone Putty should show better results and improvement in clinical outcomes such as CAL gain and PPD reduction.

## Discussion

### Expected Findings

Regenerative therapy aims to restore the original structure and functionality of the periodontal complex, including the formation of new cementum on the tooth root and new periodontal attachment between newly formed bone and cementum, as well as to regenerate periodontal attachment when periodontal disease causes the attachment apparatus to be lost [14]. The process of GTR, a dental surgical method, uses barrier membranes to guide the creation of new bone and gingival tissue when there are insufficient quantities or dimensions of bone or gingiva for appropriate function, aesthetics, or prosthetic repair. “Guided bone regeneration” is a frequent term used to describe bone-regenerative procedures or ridge augmentation. Certain cell populations in the periodontium can form a new cementum, alveolar bone, and periodontal ligament if given the opportunity to populate the periodontal wound [15]. There are 4 of these progenitor cell types: cells from the epithelium, cells produced from gingival connective tissue, cells produced from alveolar bone, and cells generated from periodontal ligaments. This method has several applications in clinical practice, including diagnosis, treatment planning, and therapeutic management [16]. GTR is a therapeutic approach that permits the restoration of bone, cementum, and periodontal ligament in degranulated osseous lesions [17]. Since its inception, GTR therapy has been applied in a variety of ways. Nonresorbable occlusal barriers were initially used to identify abnormalities of degranulated periodontal infrabony defects. Over time, the phrase “combination GTR” was adopted, and flaws were filled using bone and bone replacements before being covered with a barrier. After some time, bioresorbable barriers replaced nonresorbable ones, and biological growth factors have been used more recently to promote recovery.

Alloplastic bone graft materials are commonly used, and bioactive glass is one of them. NovaBone Putty, made from bioactive glass graft material, has recently been released. Within minutes of implantation, a special surface reaction takes place when bodily fluids come into contact with the bioactive glass substance. The first step is an ionic exchange, in which the material’s surface releases cations in return for hydrogen or hydronium ions, creating silanol groups (SiOH) [18]. Through a polycondensation reaction, silanol groups attach to one another to generate a silica-rich gel layer on the specific surface. Silica is essential for the development of bioactive glass’s bone bonding. A site for the redeposition of calcium and phosphorus from the blood and graft material is created by the silica-rich gel [19]. On top of the silica gel layer, a calcium phosphorus layer develops in a matter of hours. As the thickness and size of the layer increase over time, it transforms into a crystalline layer of hydroxycarbonate apatite (HCA), which is the same as bone material. The foundation for the link between this substance and bone is provided by this apatite layer [20]. The main benefit of bioactive glasses is their quick surface reaction rate, which promotes quick tissue attachment. The surface of the silica-rich layer is negatively charged. This raises the electrostatic charges sufficiently to accelerate the absorption of water. Water molecules and the silanol’s hydroxyl groups form hydrogen bonds, which gives bioactive glass its cohesiveness. Osteoblastic stem cells, which develop into osteoblasts and form bone, are drawn to the negatively charged surface of the HCA layer by proteins such as growth factors and fibrin, which function as an organic glue [21]. Collagen adheres to the skin and becomes a part of the HCA layer. Collagen expansion up to the junctional epithelium indirectly inhibits apical migration of the junctional epithelium. Cementum repair may be aided by bioactive glass [22].

Other characteristics of AmnioGuard, including low immunogenicity, pain relief and epithelialization promotion, self-adhesiveness, and aesthetics, have also been demonstrated. The GTR procedure is frequently carried out in conjunction with the insertion of bone grafts or bone graft replacements beneath the membrane. AM (AmnioGuard) has been used in mouth cavity repair [23]. The interconnected porous structure promotes blood vessel formation, cell occlusiveness, and biodegradability. Proliferation factors included in the AM promote the proliferation of fibroblasts, which speeds up the creation of granulation tissue [24]. Meanwhile, the AM promotes neovascularization in the surrounding tissues and vascularizes good granulation tissue [25]. Additionally, the AM offers a bioactive matrix enhanced with proteins that promotes cell motility. Therefore, it may be hypothesized that using AM as a GTR membrane could encourage cell migration and wound healing as well as vascularization of the granulation tissue in the defects. The tissue of the AM is antibacterial. β-defensins, a significant class of antimicrobial peptides secreted by epithelial cells and a crucial component of the immune system, are produced by amniotic tissue.

A concentrated mixture of growth factors obtained from platelets is known as a platelet concentrate. Four peripheral blood tubes will be taken and put into a centrifuge that has been preprogrammed. Centrifugation will be carried out using the following two procedures: (1) A-PRF with sterile plain glass–based vacuum tubes (10 mL; 1500 revolutions per minute for 14 minutes) and (2) standard PRF with sterile glass-coated plastic tubes (9 mL; 2700 revolutions per minute for 12 minutes).

### Limitations

The follow-up duration is relatively short (3‐6 months), which may not fully capture the long-term stability and success of the regenerative treatment. Future studies with extended follow-up periods are needed to evaluate the durability of the clinical and radiographic improvements observed. The study will be conducted at a single institution (Sharad Pawar Dental College), which may limit the generalizability of the findings to broader populations with diverse demographic characteristics and clinical backgrounds. Multicenter studies would help improve external validity. The participants in this study may present with varying degrees of periodontal defect severity, which could introduce heterogeneity in treatment responses and influence the observed outcomes. The success of periodontal regenerative therapy is strongly influenced by patient compliance with postoperative oral hygiene instructions and maintenance care. Variability in individual patient cooperation could impact healing and regenerative results.

### Conclusions

The combination of AmnioGuard and NovaBone Putty may be more effective in promoting bone regeneration in periodontal osseous defects than the combination of A-PRF and NovaBone Putty.

## Abbreviations

A-PRF: advanced platelet-rich fibrin
AM: amniotic membrane
CAL: clinical attachment level
GTR: guided tissue regeneration
HCA: hydroxycarbonate apatite
PPD: probing pocket depth
PRF: platelet-rich fibrin
rpm: revolutions per minute
UNC-15: 15-mm University of North Carolina probe
